# circRNA Expression Profile in Dental Pulp Stem Cells during Odontogenic Differentiation

**DOI:** 10.1155/2020/5405931

**Published:** 2020-09-01

**Authors:** Ming Chen, Yeqing Yang, Junkai Zeng, Zilong Deng, Buling Wu

**Affiliations:** ^1^Stomatological Hospital, Southern Medical University, Guangzhou, China; ^2^School of Stomatology, Southern Medical University, Guangzhou, China; ^3^Nanfang Hospital, Southern Medical University, Guangzhou, China

## Abstract

**Introduction:**

Odontogenic differentiation of human dental pulp stem cells (hDPSCs) is a key step of pulp regeneration. Recent studies showed that circular RNAs (circRNAs) have many biological functions and that competing endogenous RNA (ceRNA) is their most common mechanism of action. However, the role of circRNAs in hDPSCs during odontogenesis is still unclear.

**Methods:**

Isolated hDPSCs were cultured in essential and odontogenic medium. Total RNA was extracted after 14 days of culture, and then, microarray analysis was performed to measure the differential expressions of circRNAs. Quantitative reverse transcription-polymerase chain reaction (qRT-PCR) was then performed to validate the microarray results. Based on microarray data from this study and available in the database, a ceRNA network was constructed to investigate the potential function of circRNAs during odontogenesis. In addition, Gene Ontology (GO) and Kyoto Encyclopedia of Genes and Genomes (KEGG) analyses were performed to investigate the potential correlation between signaling pathways and circRNAs. In addition, qRT-PCR and Western blot analysis were used to explore the function of hsa_circRNA_104101.

**Results:**

We found 43 upregulated circRNAs and 144 downregulated circRNAs during the odontogenic differentiation process (fold change > 1.5 and <-1.5, respectively; *P* < 0.05). qRT-PCR results were in agreement with the microarray results. Bioinformatic analysis revealed that the Wnt signaling pathway and the TGF-*β* signaling pathway, as well as the other pathways associated with odontogenic differentiation, were correlated to the differentially expressed circRNAs. hsa_circRNA_104101 was proved to promote the odontogenic differentiation of hDPSCs.

**Conclusion:**

This study reported 187 circRNAs that were differentially expressed in hDPSCs during odontogenic differentiation. Bioinformatic analysis of the expression data suggested that circRNA-miRNA-mRNA networks might act as a crucial mechanism for hDPSC odontogenic differentiation, providing a theoretical foundation for the study of pulp regeneration regulation by circRNAs.

## 1. Introduction

Pulpal and periapical diseases are the most common oral diseases: the traditional treatment of choice is root canal therapy. However, the excessive loss of pulp tissue could increase the risks of fracture and the subsequent failure of the root canal therapy, resulting in serious adverse effects, including intraradicular microorganisms, extraradicular infection, foreign body reaction, and true cysts [1]. Thanks to the advancements of tissue engineering, regenerative medicine, and material science, pulp regeneration will become the new desirable standard, particularly the regeneration of the dentin-pulp complex with biological techniques [2]. The regeneration requires three essential prerequisites: seed cells, cytokines, and scaffolds [3, 4]. In recent years, a variety of stem cells have been isolated from oral tissues, such as human dental pulp stem cells (hDPSCs) [5], stem cells from apical papilla (SCAPs) [6], periodontal ligament stem cells (PDLSCs) [7], dental follicle progenitor cells (DFPCs) [8], and stem/progenitor cells isolated from the human pulp of exfoliated deciduous teeth (SHED) [9]. hDPSCs are the most common dental-derived stem cells used in the study of odontogenic differentiation [10]: they originate from neural crest mesenchymal stem cells and play an important role in pulp regeneration, thanks to their multiple differentiation potential and self-renewal capacity [11, 12]. A previous study reported the differentiation of pulp-like tissues in a subcutaneous immunodeficient mouse model, after the implantation of human tooth root segments with 3D-DPSCs [2]. Although many groups have been studying the odontogenic differentiation [13–15], the exact molecular mechanisms are still poorly understood, and thus, the regeneration of dentin-pulp complex in the clinical context is still not implemented [16]. Therefore, the search for new biomarkers linked to the molecular mechanisms of odontogenic differentiation is extremely important for studying pulp regeneration.

In recent years, research on noncoding RNA (ncRNA) has gradually become more prominent. Circular RNA (circRNA) is a family of ncRNA characterized by a cyclic covalent structure. Compared with linear RNA, its cyclic structure confers resistance to RNase R digestion [17, 18]. Thus, circRNAs are not easily degraded and are more stable than linear RNA [19]. In addition, circRNAs are widely distributed in the organism and have a high evolutionary conservation and tissue cell expression specificity [20]. In some cases, the abundance of circRNA exceeds the abundance of the related linear mRNA by a factor of 10 [21, 22]. Therefore, circRNAs are considered ideal diagnostic markers and therapeutic targets, with very important research significance and clinical value [21, 22].

Some studies have found that microRNAs (miRNAs) may act as negative regulators of mRNA translation through the binding to the 3′-UTR mRNA region. Intriguingly, thanks to the presence of miRNA binding sites, circRNAs can function as competing endogenous RNA (ceRNA) by sequestering the relative miRNAs, inhibiting their function [23]. It has been already reported that circRNAs play an important role in the regulation of pluripotent dental stem cell differentiation, but these studies were focused on oral maxillofacial tumors and periodontal diseases [24, 25]. Moreover, a previous research found that the circRNA CDR1as promoted the osteoblastic differentiation of periodontal ligament stem cells by downregulating miR-7 and concurrently stimulating the expression of GDF5, which is usually inhibited by miR-7. GDF5 then activates the Smad1/5/8 and p38 MAPK differentiation pathway [26]. In the same year, Wang et al. found that the circRNA DOCK1, functioning as ceRNA, can regulate the expression of BIRC3, a protein involved in apoptosis in oral squamous cell carcinomas (OSCC) [25]. In addition, Li et al. suggested that the circRNA circNTATC1 could promote the osteogenic differentiation of apical papilla stem cells. By using CircBase, they predicted that the miRNA miR-4483 contains binding sites for circNFATC1 and that the expression of circNFATC1 is negatively correlated with miR-4483 expression [27]. However, now, the effects of circRNAs on hDPSC odontogenic differentiation are unclear. Thus, studying the molecular mechanisms of circRNA function can provide new biomarkers and a potential target for the regenerative treatment using mesenchymal stem cells.

In this paper, we analyzed the differential expression of circRNAs in hDPSCs during odontogenic differentiation by using microarray technology. Then, we validated the top 6 differentially expressed circRNAs by qRT-PCR. Using available data on miRNAs, TargetScan, miRDB, CircBase, CircBank, and other databases, we performed a bioinformatic analysis to construct a circRNA-miRNA-mRNA network of odontogenic gene regulation. Next, we performed Gene Ontology (GO) and Kyoto Encyclopedia of Genes and Genomes (KEGG) analyses to detect the association between circRNA differential expression and pathways associated with odontogenic differentiation. Finally, hsa_circRNA_104101 was selected for further research according to the bioinformatic analyses, and it might be involved in the process of odontogenic differentiation of hDPSCs through sponge effect. Our study was aimed at providing a theoretic foundation for elucidating the circRNA molecular mechanism of hDPSCs during odontogenic differentiation.

## 2. Materials and Methods

### 2.1. Cell Culture and Identification

hDPSCs were isolated from the teeth acquired from 18-25-year-old patients who have undergone tooth extraction at the Department of Stomatology of Nanfang Hospital, Southern Medical University, Guangzhou, Guangdong, China. All experimental protocols were approved by the Ethical Committee of Southern Medical University. Culturing programs of hDPSCs were the same as described previously [28]. Then, hDPSCs were cultured in Dulbecco's modified Eagle's medium (DMEM) added with 10% fetal bovine serum (FBS; GIBCO, Life Technologies, Australia), 100 U/ml penicillin, and 100 *μ*g/ml streptomycin (Sigma, St. Louis, Mo, USA) at 37°C under 5% CO_2_ in air. The culture media were renewed every 3 days. hDPSCs of passages 3–5 were used in follow-up experiments [29, 30], respectively, cultured in essential and odontogenic medium. hDPSCs in the noninduced were cultured in 10% FBS in DMEM with no supplements. hDPSCs in the induced group were cultured with an odontogenic differentiation medium containing 50 mg/ml ascorbic acid, 100 nmol/l dexamethasone, and 10 mmol/l *β*-glycerophosphate (Sigma, St Louis, Mo, USA) in DMEM for 14 days [31, 32]. The culture media were renewed every 3 days.

Odontogenic, adipogenic, and chondrogenic differentiation was performed to identify the multiple differentiation potential of hDPSCs. Alizarin red, oil red O, and Alcian blue staining is commonly used to verify the formation of calcified nodules, lipid droplets, and mucopolysaccharides after stem cell osteogenic, adipogenic, and chondrogenic differentiation [32–34]. When hDPSCs were in the 3rd generation, logarithmic growth phase, we removed the complete original medium, washed cells with PBS buffer for 2-3 times, and digested them with 0.25% trypsin to collect the cell suspension and then inoculated into 6-well plates, 1 × 10^5^ cells/well. The fresh medium was replaced every 3 days. When the cells proliferated to a density of 80%, the cells were replaced with odontogenic (contained with 10% FBS, 50 mg/ml ascorbic acid, 100 nmol/l dexamethasone, and 10 mmol/l *β*-glycerophosphate), adipogenic (contained with 10% FBS, 0.5 mM isobutyl-methylxanthine (IBMX), 10−6 M dexamethasone, 10 *μ*g/ml insulin, and 200 *μ*M indomethacin), and chondrogenic (contained with 10% FBS, 1% ITS+Premix, 0.1 *μ*M dexamethasone, 0.2 mM L-ascorbic acid-2-phosphate, 1% pen/strep, and 10 ng/ml TGF-*β*3) induction medium, and the cells were placed in a 37° C, 5% CO_2_ cell culture incubator. The culture media were renewed every 3 days.

Flow cytometry was used to identify the cell phenotypes of hDPSCs. hDPSCs at the 3rd passage were isolated by trypsin, adjusted to a concentration of 1 × 10^6^/ml/well. The cells were conjugated for surface markers of hematopoietic cells (CD45, CD34) and mesenchymal stem cells (Stro-1, CD90, CD44, and CD29) according to protocol and then were detected by flow cytometry. hDPSCs at the 3rd passage with a concentration of 100 cells/well were inoculated into 6-well plates and cultured for 14 days. After that, the cells were fixed with 4% paraformaldehyde and stained with crystal violet stain.

### 2.2. RNA Extraction

Total RNAs were extracted from induced and noninduced hDPSCs with a TRIzol reagent (Invitrogen, Life Technologies) based on the manufacturer's protocol. Total RNA was quantified by NanoDrop ND-1000 (Thermo Scientific, USA).

### 2.3. circRNA Microarray

After extraction and purification, the total RNAs of the induced and noninduced groups (three cell samples per group) were analyzed by using microarray. Arraystar Human circRNA Array (Arraystar, USA) was used to perform RNA expression profiling. After RNAs were digested with RNase R, a random priming method (Arraystar Super RNA Labeling Kit; Arraystar) was used for the amplification and transcription of circRNAs to transfer into fluorescent cRNA. The arrays were analyzed by the Agilent Scanner G2505C, and Agilent Feature Extraction software (version 11.0.1.1) was used to access obtained array images.

### 2.4. Bioinformatic Analysis

In order to understand the potential functions of the differentially expressed circRNAs that were statistically significant (*P* < 0.05; fold change > 1.5 or <-1.5), miRanda and TargetScan software were used to predict the putative circRNA/miRNA interactions. Then, potential target genes of miRNAs were predicted using the TargetScan, miRDB, CircBase, and CircBank databases. Both GO analysis and KEGG were carried out. The GO project is able to represent the characterization of gene and gene product in all organisms. The ontology contains cellular component, biological process, and molecular function. To predict the pathways, KEGG was performed to sort differentially expressed genes on the basis of the KEGG database. The ceRNA networks were constructed with circRNAs, miRNAs, and mRNAs. Cytoscape 3.5.1 was used to reveal the networks of circRNA-miRNA-mRNA.

### 2.5. Identification of hsa_circRNA_104101

Agarose gel electrophoresis and Sanger sequencing were required to verify hsa_circRNA_104101. According to the sequence information of hsa_circRNA_104101, we designed the divergent primer and the convergent primer (Table 1). cDNA (complementary DNA) and gDNA (genomic DNA) were extracted from induced and noninduced hDPSCs based on the manufacturer's protocol. Then, cDNA and gDNA were, respectively, for PCR amplification, and the amplified products were obtained by agarose gel electrophoresis. The PCR products of hsa_circRNA_104101 in the cDNA were sent to the company for Sanger sequencing.

### 2.6. Lentivirus Construction and Infection

The siRNA-104101 (divided into 1, 2, and 3 strands) (Table 2) and siRNA-control were used to carry out instantaneous gene knockdown of hDPSCs using liposome as the carrier. At 72 h after transfection, the expression level of hsa_circRNA_104101 after gene knockdown was detected by qRT-PCR. Next, the second passages of hDPSCs were selected for experiments and construction of hsa_circRNA_104101 shRNA lentivirus. Finally, we need to determine the MOI of lentivirus-infected cells and the optimal infection conditions.

### 2.7. Alkaline Phosphatase and Alizarin Red Staining

Alkaline phosphatase (ALP) activity staining was performed using the NBT/BCIP Staining Kit (Beyotime Biotech, Shanghai, China); Alizarin red staining was showed to visualize mineral deposition in each group. Cells were washed with PBS for three times and were fixed in 4% paraformaldehyde for 15 mins. After washing, the hDPSCs were stained with the NBT/BCIP Staining Kit and Alizarin red. The results of each group were photographed under an inverted microscope.

### 2.8. qRT-PCR Validation

Total RNAs were isolated from two groups of hDPSCs. 1 *μ*g of RNA per sample was reverse transcribed into cDNA using a cDNA Reverse Transcription Kit (Takara, Tokyo, Japan). Divergent primers were used for analysis of circRNAs. qRT-PCR was performed in a 20 *μ*L of the reaction system, and glyceraldehyde-3-phosphate dehydrogenase (GAPDH) was used as an internal control on the Roche LightCycler®480 sequence detection system. All experiments were performed in triplicate, and relative gene expression was represented as fold change using the 2^-*ΔΔ*Ct^ method. A value of *P* < 0.05 was considered statistically significant [35]. The sequences of gene-specific primers are listed in Table 3.

### 2.9. Western Blot Analysis

Total protein was isolated from hDPSCs using the RIPA lysis buffer and quantitated using the BCA assay. The protein lysates were separated by 10% SDS-PAGE, followed by transferring onto PVDF membranes. Primary antibodies against DSPP, DMP1, ALP, OCN, and GAPDH were diluted at 1 : 1000. After blockage in protein free rapid blocking buffer for 20 min, the PVDF membranes were incubated with primary antibodies overnight at 4°C. Next, PVDF membranes were incubated with corresponding secondary antibodies for 1 h at room temperature. Immunoreactive proteins were detected by using the ECL Kit (Beyotime Biotech, Shanghai, China), and the gray value of protein bands was calculated by ImageJ software.

### 2.10. Statistical Analysis

All statistical analyses were performed by using SPSS 19.0 software. Quantitative data were presented as the mean ± standard deviation from three independent records. Student's *t*-test was used to calculate the statistical significance. *P* < 0.05 was considered statistically significant.

## 3. Results

### 3.1. Identification and Odontogenic Differentiation of hDPSCs

**Figure 1:**
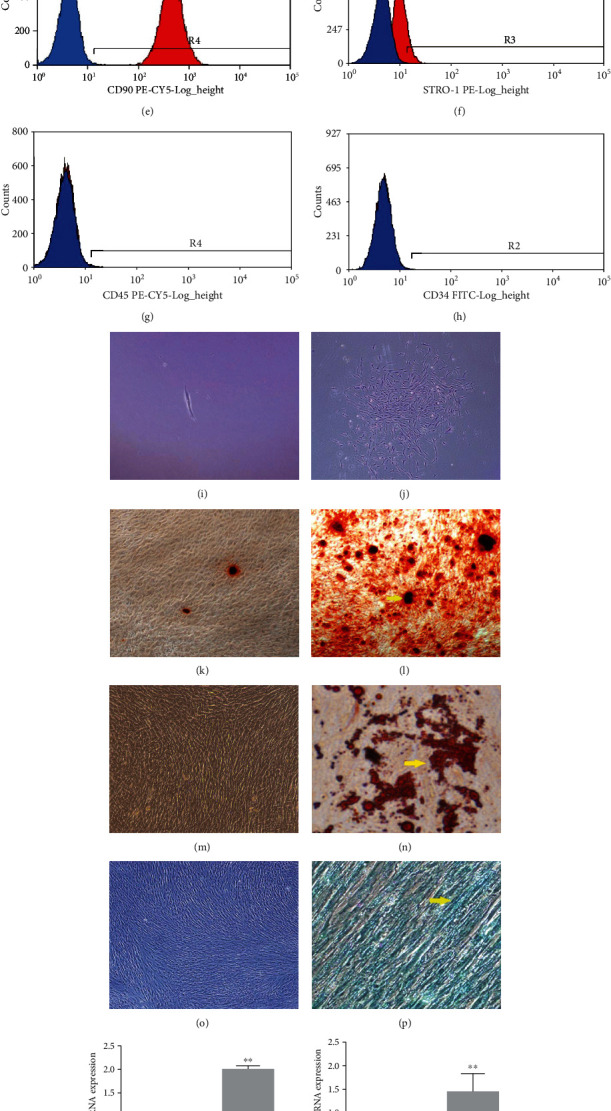
Identification and multiple differentiation potential of hDPSCs. (a) hDPSCs were separated from dental pulp. (b) hDPSCs were subcultured with normal media at third passage. (c–h) hDPSCs were positive for Stro-1 (18.39%), CD44 (99.99%), CD29 (97.96%), and CD90 (99.99%) and negative for CD45 (0.46%) and CD34 (0.35%). (i, j) A single cell was obtained, and single cell-derived colonies were obtained after culturing for 14 days. (k, 1) Noninduced group and induced group of odontogenic differentiation. Mineral nodes formed in the induced group were as indicated by the yellow arrow. (m, n) Noninduced group and induced group of adipogenic differentiation. Lipid droplets formed in the induced group were indicated by the yellow arrow. (o, p) Noninduced group and induced group of chondrogenic differentiation. Expressed glycosaminoglycans formed in the induced group were indicated by the yellow arrow. (q–s) In the induced group, upregulated expressions of odontogenic differentiation-related genes DSPP, DMPI, and OCN by qRT-PCR were shown, compared with the noninduced group. All samples were performed in triplicate. The data are represented as means ± SD. ^∗^*P* < 0.05, ^∗∗^*P* < 0.01.

hDPSCs extracted from dental pulp were cultured to the 3rd passages in normal media (Figures 1(a) and 1(b)). The results of flow cytometry revealed that hDPSCs could express mesenchymal stem cell markers CD44 (99.99%), CD29 (97.96%), CD90 (99.99%), and Stro-1 (18.39%) but barely express hematopoietic cell markers including CD45 (0.46%) and CD34 (0.35%) (Figures 1(c)–1(h)). A single cell was obtained by limiting dilution technique, and clone formation was observed after 14 days of single cell culture (Figures 1(i) and 1(j)). Staining of Alizarin red, oil red O, and Alcian blue was used to confirm multipotency of hDPSCs after 21 days of induction (Figures 1(k)–1(p)). Subsequently, the qRT-PCR results suggested that the expressions of odontoblastic markers dentin sialophosphoprotein (DSPP), dentin matrix acid phosphoprotein 1 (DMP1), and osteocalcin (OCN) were upregulated. Our findings agree with the previous study reporting the differentiation of hDPSCs into odontoblasts.

### 3.2. Expression Profiles of circRNA during Odontogenic Differentiation

**Figure 2:**
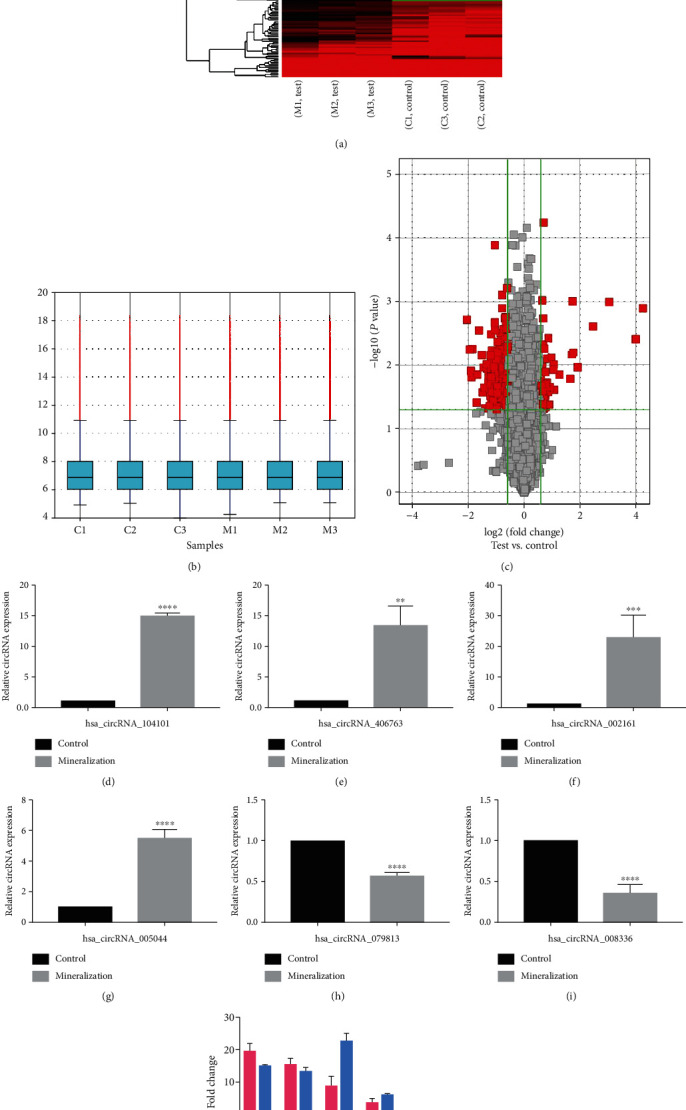
Differentially expressed circRNAs between different groups, differentially expressed circRNAs validated by qRT-PCR. (a) Hierarchical cluster analysis of circRNAs in the induced and noninduced groups by gene chip. The parts of red and green, respectively, mean the upregulation and downregulation of circRNA. (b) Box plot indicated that the distribution of the intensities among all samples was nearly the same. (c) Red font in the volcano plot denotes the differentially expressed circRNAs with statistical significance from hDPSCs between the induced and noninduced groups (fold change > 1.5 or <-1.5, *P* < 0.05). (d–g) circRNA hsa_circRNA_104101, hsa_circRNA_406763, hsa_circRNA_002161, and hsa_circRNA_005044 were upregulated in the induced group in comparison with the noninduced group. (h, i) circRNAs hsa_circRNA_079813 and hsa_circRNA_008336 were downregulated. The data were represented as means ± SD. ^∗^*P* < 0.05, ^∗∗^*P* < 0.01, ^∗∗∗^*P* < 0.001, and ^∗∗∗∗^*P* < 0.0001. (j) Results of the comparison of microarray and qRT-PCR data for candidate circRNAs.

The expression profiles of 12929 human circRNAs of hDPSCs during odontogenic differentiation were obtained by the use of circRNA microarray. The heatmap analysis revealed that the inner-group variation was small, suggesting that these alterations may be common in the odontogenic differentiation of hDPSCs (Figure 2(a)). The box plot suggests that the distribution of the intensities among the whole samples was practically consistent (Figure 2(b)). As can be seen from the volcano plots, we found that compared with the noninduced group, 187 circRNAs displayed beyond a 1.5-fold differential expression in the induced group, and 44 circRNAs were upregulated while 143 circRNAs were downregulated (Figure 2(c)). The details of differentially expressed circRNAs are shown in Tables S1 and S2. Among these circRNAs with statistical significance, most of them are distributed on human chromosome2, chromosome6, and chromosome7, of which the upregulated circRNAs are mainly distributed on chromosome2, while the downregulated circRNAs are mainly distributed on chromosome6 and chr7. circRNAs can be divided into three types: exon, intron, and sense overlapping, but most of these circRNAs were mapped to exons.

### 3.3. qRT-PCR Validation of circRNA Expression

To confirm the results of the microarray data experiments, qRT-PCR analysis was performed on induced and noninduced hDPSCs, respectively. Compared with the noninduced group, hsa_circRNA_104101, hsa_circRNA_406763, hsa_circRNA_002161, and hsa_circRNA_005044 were upregulated by 19.03, 15.95, 8.29, and 3.78-fold, respectively, in the induced group. The expressions of hsa_circRNA_079813 and hsa_circRNA_008336 were downregulated by 3.85 and 3.74-fold, respectively. Among the 187 circRNAs with significantly different expressions before and after mineralization induction, these 6 circRNAs showed the most significant changes, and primers with good specificity could be designed for them. Therefore, these six differentially expressed circRNAs were selected for verification, and all qRT-PCR results were in the agreement with the normalized expression of microarray data (Figures 2(d)–2(j)).

### 3.4. circRNA-miRNA-mRNA Network

**Figure 3:**
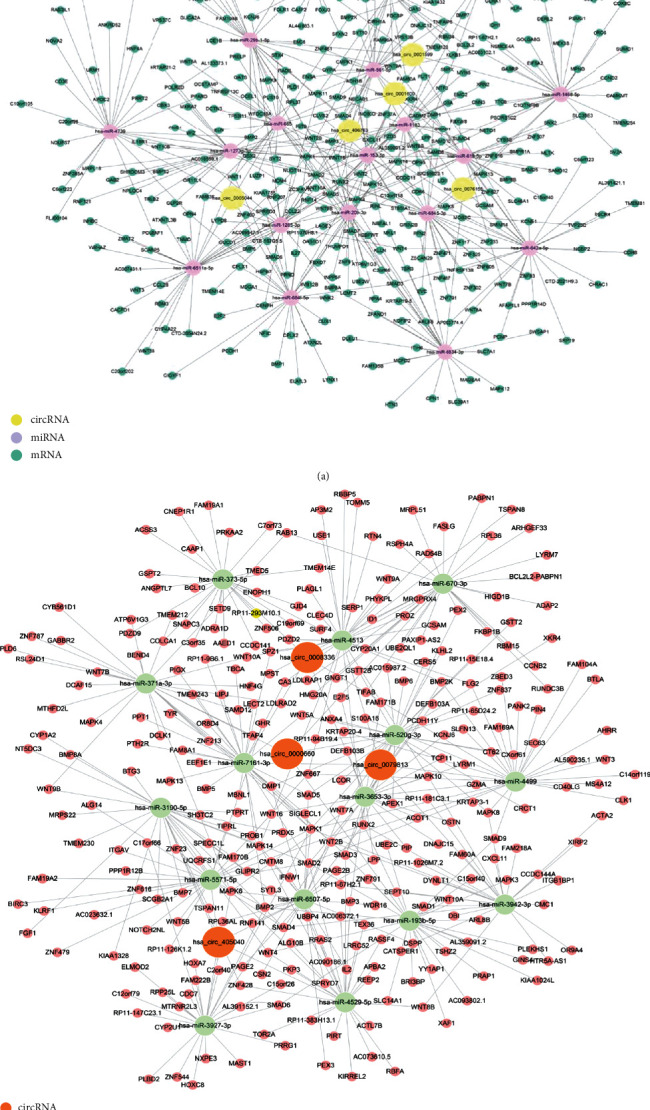
circRNA-miRNA-mRNA network. Several most significantly different circRNAs were selected from the results of the gene chip, miRanda, and Targetscan software which were used to determine the circRNAs combined with miRNAs, and TargetScan, miRDB, CircBase, and CircBank were used to predict target mRNAs. (a) CeRNA networks of upregulated circRNAs-miRNAs-mRNAs, included 5 circRNAs, 19 miRNAs, and 363 mRNAs. Yellow circle represents circRNAs, purple circle represents miRNAs, green circle represents underlying target mRNAs, and the lines indicate interactions between circRNAs and miRNAs or miRNAs and mRNAs. (b) CeRNA networks of downregulated circRNAs-miRNAs-mRNAs, included 4 circRNAs, 16 miRNAs, and 258 mRNAs. Orange circle represents circRNAs, light green circle represents miRNAs, pink circle represents underlying target mRNAs, and the lines indicate interactions between circRNAs and miRNAs or miRNAs and mRNAs.

Analysis of 187 significant differences (fold change > 1.5 or <-1.5, *P* < 0.05) in circRNAs based on biological information will have a number of potential regulatory mechanisms. Then, we selected several circRNAs from the circRNAs with significant differences for further research to construct circRNA-miRNA-mRNA networks, including 5 circRNAs from upregulated genes and 4 from downregulated genes. First of all, miRanda and TargetScan software were used to determine the circRNAs combined with miRNAs, and TargetScan, miRDB, CircBase, and CircBank were used to predict target mRNAs. Then, we evaluated the miRNA response elements (MREs) related to circRNAs to verify all of the circRNA-miRNA connections. The miRNAs were ranked based on their mirSVR scores, and the top 5 miRNAs for each circRNA were selected for further analysis. We predicted and selected 25 corresponding target genes for each miRNA based on transcriptome from microarray results. After that, 2 ceRNA networks were built by the use of Cytoscape: one network constructs with downregulated circRNAs, miRNAs, and mRNAs (fold change < −1.5; *P* < 0.05); the other one consists of upregulated circRNAs, miRNAs, and mRNAs (fold change > 1.5; *P* < 0.05) (Figures 3(a) and 3(b)).

### 3.5. GO and KEGG Pathway Analysis

**Figure 4:**
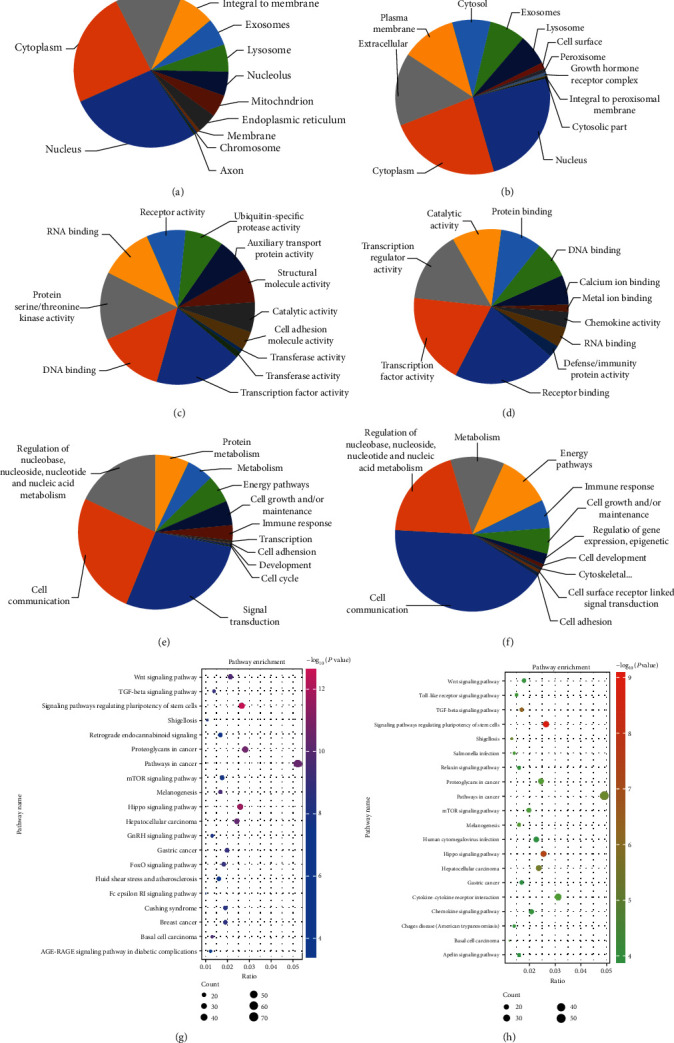
GO and KEGG pathway analysis of noninduced and induced hDPSCs. GO analysis of 1345 upregulated differentially expressed mRNAs and 1058 differentially expressed downregulated mRNAs, respectively. (a) GO cellular component classification of upregulated mRNAs. (b) GO cellular component classification of downregulated mRNAs. (c) GO molecular function classification of upregulated mRNAs. (d) GO molecular function classification of downregulated mRNAs. (e) GO biological process classification of upregulated mRNAs. (f) GO biological process classification of downregulated mRNAs. KEGG pathway enrichment. The *X*-axis represents the ratio of enriched differentiated genes to all genes enriched for a particular pathway term. Spot size represents the number of significantly differentiated genes; larger spots indicate more differentiated genes; the color of circle indicates the *P* value including the signaling pathways regulating pluripotency of stem cells, the Wnt pathway, and the TGF-*β* pathway. (g) KEGG pathway analysis of upregulated mRNAs. (h) KEGG pathway analysis of downregulated mRNAs.

To investigate the biological functions of the dysregulated circRNAs, we selected the top five predicted miRNAs for each circRNA through specific base pairing. We performed GO and KEGG pathway analysis with their target genes. GO analysis results showed that the enriched GO terms for the biological process were cell communication, signal transduction, and regulation of nucleobase and nucleotide. Through cellular component analysis, the target genes were widely involved in the composition of the nucleus, cytoplasm, plasma membrane, etc. The molecular function structured networks indicate that the circRNA target genes can participate in a variety of molecular functions, including DNA binding, transporter activity, and receptor activity (Figures 4(a)–4(f)). With the latest KEGG database, pathway analysis was performed to identify the biological functions of circRNAs and correlate the differentially expressed circRNAs with corresponding target genes. These differential genes were enriched in signaling pathways regulating pluripotency of stem cells, Wnt signaling pathway, TGF-*β* signaling pathway, and some other signaling pathways associated with the differentiation of hDPSCs to odontogenic cells (Figures 4(g) and 4(h)).

### 3.6. Identification of hsa_circRNA_104101

**Figure 5:**
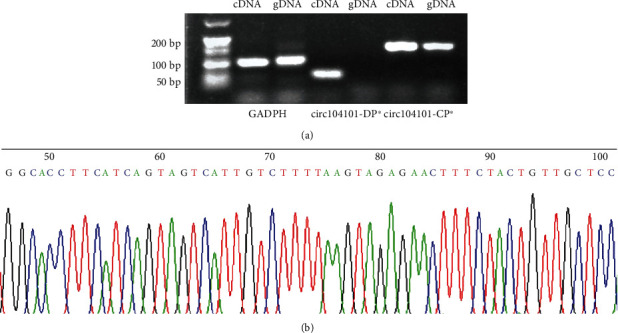
Identification of hsa_circRNA_104101. (a) Agarose gel electrophoresis experiment. ∗: DP: divergent primer; CP: convergent primer. (b) Sanger sequencing.

The results of agarose gel electrophoresis showed that the amplified products of all two primers were single bands, and the bands were consistent with the primer amplification length. What is more, a set of convergent primer can amplify not only gDNA but also cDNA; divergent primer amplified circRNA in cDNA but not in gDNA (Figure 5(a)). The result of Sanger sequencing was consistent with the back-splice junction of hsa_circRNA_104101 (Figure 5(b)).

### 3.7. Construction of hsa_circRNA_104101 shRNA Lentivirus and Cell Infection

**Figure 6:**
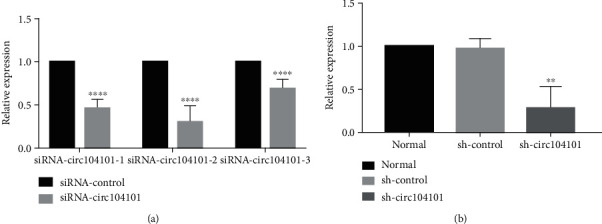
Construction of hsa_circRNA_104101 shRNA lentivirus and cell infection. (a) siRNA cell transfection (the data were represented as means ± SD for each group: ^∗∗∗^*P* < 0.001, ^∗∗∗∗^*P* < 0.0001). (b) Knockdown of hsa_circRNA_104101 after lentivirus transfection was confirmed by qRT-PCR (the data were represented as means ± SD for each group, ^∗∗^*P* < 0.01).

It was found that all three designed siRNA-104101 could downregulate the expression of hsa_circRNA_104101 in hDPSCs, and the results of all siRNA were statistically significant (*P* < 0.01) (Figure 6(a)), among which the second strand was the most downregulated with the most obvious results. According to the results of siRNA transfection, sh-circ104101 was designed with the second strand, and hDPSCs were transfected with the best infection condition (MOI:30 and HiTransG P). The second-generation hDPSCs were obtained and divided into three groups: normal group, sh-control group, and sh-circ104101 group. The results of qRT-PCR showed that there was no significant difference between the normal group and the sh-control group (*P* > 0.05). Compared with the sh-control group, the expression of the sh-circ104101 group decreased significantly (*P* < 0.01) (Figure 6(b)).

### 3.8. The Effect of hsa_circRNA_104101 on the Odontogenic Differentiation of hDPSCs

**Figure 7:**
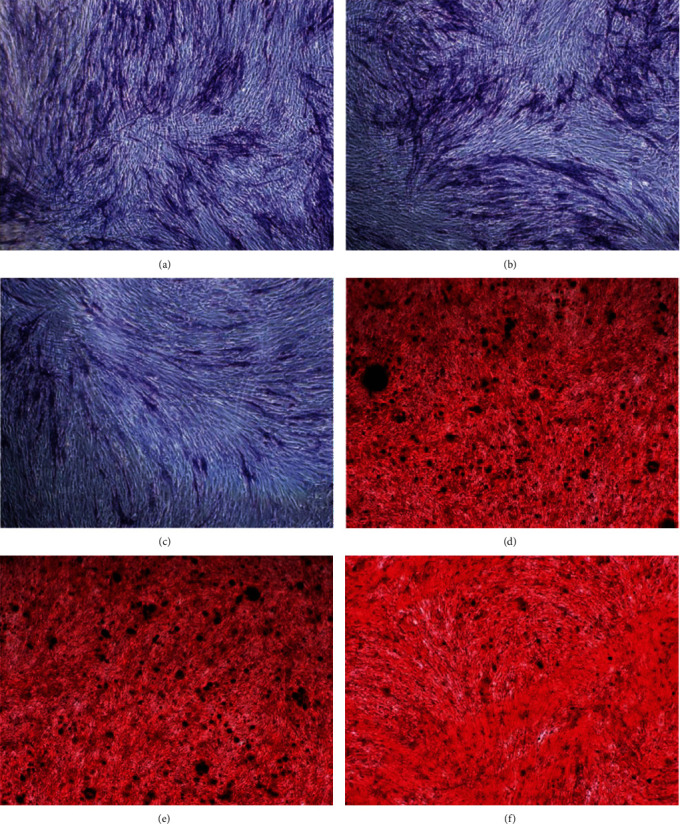
ALP activity examination and Alizarin red staining. (a) ALP staining in normal group. (b) ALP staining in sh-control group. (c) ALP staining in sh-circRNA group. (d) ARS staining in normal group. (e) ARS staining in sh-control group. (f) ARS staining in sh-circRNA group.

**Figure 8:**
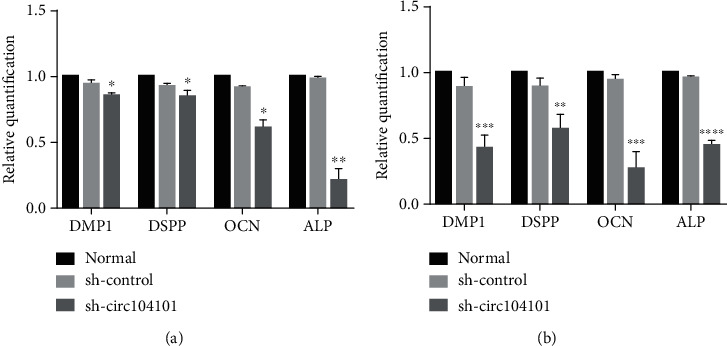
Expression of mineralization-related mRNA after hDPSC transfection. (a) The expression level of DMP1, DSPP, OCN, and ALP decreased in the sh-circ104101 group at 7 days after odontogenic differentiation using qRT-PCR. (b) The expression level of DMP1, DSPP, OCN, and ALP decreased in the sh-circ104101 group at 14 days after odontogenic differentiation using qRT-PCR (the data were represented as means ± SD for each group: ^∗^*P* < 0.05, ^∗∗^*P* < 0.01, ^∗∗∗^*P* < 0.001, and ^∗∗∗∗^*P* < 0.0001).

**Figure 9:**
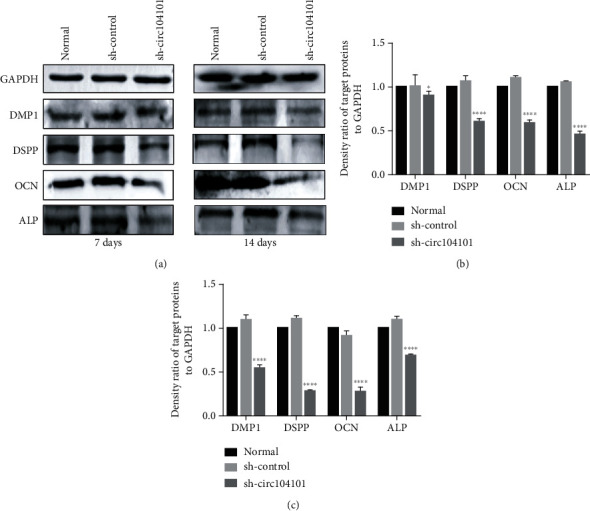
Expression of mineralization-related proteins after hDPSC transfection. (a) Western blot analysis shows the expression level of DMP1, DSPP, OCN, and ALP decreased in the sh-circ104101 group after odontogenic differentiation for 7 and 14 days. GAPDH was used as an internal control. The data were represented as means ± SD for each group: ^∗^*P* < 0.05, ^∗∗∗∗^*P* < 0.0001, compared with the differentiated sh-control group. (b, c) The density ratio of target proteins to GAPDH for 7 and 14 days.

The staining of ALP decreased in the sh-circ104101 group compared with the normal and sh-control groups after induction for 7 days. Moreover, the contents of mineralized nodule formation of Alizarin red staining decreased in the sh-circ104101 group after odontogenic induction for 14 days (Figure 7). In addition, qRT-PCR was used to detect the expression of mineralization-related factors in the normal group, the sh-control group, and the sh-circ104101 group after odontogenic induction for 7 and 14 days, respectively. The mRNA expressions of DSPP, DMP1, ALP, and OCN were downregulated after hsa_circRNA_104101 knockdown in hDPSCs (Figure 8). Western blot results showed that the protein levels of DSPP, DMP1, ALP, and OCN were also decreased in the sh-circ104101 group (Figure 9).

## 4. Discussion

circRNA molecules are more structurally stable than linear RNA [19]. In addition, circRNAs are widely distributed and have a high evolutionary conservation and tissue expression specificity [20]. In some cases, the circRNA has an abundance of more than 10 times compared to the relative linear mRNA. Therefore, circRNAs are considered to be the ideal disease diagnostic marker and therapeutic target and have a great potential clinical value [21, 22]. In recent years, many investigators demonstrated that long noncoding RNA (lncRNA), miRNA, and other ncRNAs are involved in the hDPSC odontogenic differentiation. However, the role of circRNA in this process was poorly understood. In our study, the expression of 12929 circRNAs was detected during hDPSC odontogenic differentiation using microarray analysis. For about 1% of them (187), we found a significantly different expression in the differentiated state compared to the undifferentiated state (fold change > 1.5 or <-1.5). Of the 187 differently expressed circRNAs, 43 were upregulated and 144 downregulated. The most significantly upregulated circRNAs are transcribed from exons (19-fold change). This suggests that circRNA may play a significant role in the odontogenic differentiation of hDPSCs.

To further understand the potential regulatory role of the differentially expressed circRNAs, we performed KEGG pathway and GO analysis to evaluate which cellular functions involved the circRNA target genes. In the GO analysis, we found that the target genes were correlated with many important biological processes, including odontogenic differentiation. In particular, the more represented functional classes were the following: cellular communication and signal transduction. By performing the KEGG analysis, we predicted that the target genes were correlated with the Wnt pathway, TGF-*β* pathway, and other signaling pathways regulating stem cell pluripotency. This is in agreement with a previous study that reported that the Wnt signaling pathway has an important role in odontoblast biology [36]. Moreover, it is already known that ncRNAs can inhibit the odontogenetic differentiation of hDPSCs through Wnt signaling pathway inhibition [32]. Additionally, the TGF-*β* pathway is essential in the repair of pulp injury: when dentin is damaged, the dentin matrix releases TGF-*β*1, which can induce dentin cells to regenerate to repair the tissue [37]. Thus, our results suggest that the differentially expressed circRNAs detected in this study regulate odontogenic differentiation through these two pathways.

It has been shown that circRNA plays an important role in the osteogenic differentiation of a variety of stem cells. However, the research on the role of circRNA in the odontogenic differentiation of hDPSCs only focuses on the analysis of high-throughput sequencing results and bioinformatics [38]. Therefore, we selected one predicted circRNA based on our results for subsequent functional experiments. In order to study the effect of hsa_circRNA_104101 on the odontogenic differentiation of hDPSCs, we carried out the hsa_circRNA_104101 RNA interference silencing experiment. RNA interference can specifically knock down the target gene expression, which is the most widely used technology to study the biological function of a gene. In our experiment, sh-circ104101 was successfully synthesized and packaged into lentivirus, which successfully inhibited the expression of hsa_circRNA_104101. After 14 days of mineralization induction, the odontogenic differentiation-related genes and proteins were detected by qRT-PCR and Western blot. It was found that the expression of DSPP, DMP1, ALP, and OCN decreased in varying degrees. DSPP is an important noncollagenous protein in the dentin matrix, which is mainly expressed in odontoblasts, and is closely related to tooth development and biomineralization. DMP1 is a noncollagenous extracellular matrix protein of dentin, which plays an important role in the odontogenic differentiation of undifferentiated mesenchymal cells [23]. ALP is considered to be an important marker in the early stage of osteogenesis, which promotes calcification in the formation of hard tissue. OCN is one of the differentiation markers from osteoblasts to mineralization stage. At the same time, Alizarin red staining showed that the formation of calcium nodules was reduced. The above results indicated that the silencing of hsa_circRNA_104101 could inhibit the odontogenic differentiation of hDPSCs, which provided the experimental basis for the follow-up mechanism study, and also provided a reference for the role of other circRNAs in the process of odontogenic differentiation of hDPSCs.

Several gene chip data results available from different databases were used for constructing a circRNA-miRNA-mRNA network. Multiple miRNAs and mRNAs can be regulated by a singular circRNA through different mechanisms, revealing a complex interaction between these molecules. Interestingly, some of the miRNAs detected in this network have been already demonstrated to regulate differentiation processes. For instance, it has been reported that, in peripheral blood monocytes, miR-708-5p can negatively regulate the expression level of factors involved in bone metabolism such as AKT1, AKT2, PARP1 FKBP5, and MP2K3, resulting in an osteoporotic phenotype [39]. Moreover, the lncRNA HIF1A-AS2 can competitively bind miR-665 and thus positively regulates IL6, activating the PI3K/Akt signaling pathway, which promotes osteogenic differentiation of adipose-derived stem cells [40]. Overexpression of miR-665 was found to decrease the recruitment of the negative regulators KAT6A to DSPP and DMP1 gene promoters during dentinogenesis [41]. Elsafadi et al. reported that miR-4739 overexpression could inhibit the osteogenic differentiation of human bone marrow stromal cells [42]. In our ceRNA network, we predicted that the odontogenic differentiation-related genes DSPP and DMP1 might be correlated to has-miR-708-5p and has-miR-642a-5p. In turn, these miRNAs may be correlated to the differentially expressed circRNAs during hDPSC odontogenic differentiation, although these potential novel regulatory pathways should be experimentally confirmed in future studies.

It is generally accepted that stem cells have various differentiation potential, which is regulated by the presence of different signal molecules and bioactive materials. In the context of odontogenic differentiation, stem cells differentiate into pulp, dentin, and other cell types [43]. Pulp revascularization has been widely used for the treatment of pulp necrosis. However, it was extensively reported that the tissues in the root canals consist of cementum-like, bone-like, and periodontal ligament-like tissues [44, 45]. Induced repair of dental pulp is probably different from the real physical regeneration [46, 47].

Therefore, the follow-up experiments of our study were mainly focused on whether hsa_circRNA_104101 can regulate the mineralization process as a ceRNA function. We first intend to further utilize fluorescence in situ hybridization to locate hsa_circRNA_104101, then identify miRNA molecules interacting with circRNA by RNA pull-down experiment, and detect specific binding sites by a dual luciferase reporter system to further analyze its mechanism.

## 5. Conclusion

In summary, we found that 187 circRNAs were differentially expressed in hDPSCs during odontogenic differentiation. Moreover, we performed a bioinformatic analysis to detect pathways linked to odontogenic differentiation through circRNAs. We predicted that the Wnt and TGF-*β* signaling pathway, as well as the other pathways associated with odontogenic differentiation, are correlated to the differentially expressed circRNAs. We constructed a circRNA-miRNA-mRNA network, which may provide a new theoretical foundation for the study of pulp regeneration. Our results unveil the potential role of circRNA in odontogenic differentiation. In addition, our study indicated that hsa_circRNA_104101 was involved in the odontogenic differentiation of hDPSCs. circRNAs could be novel therapeutic targets for pulp regeneration.

## Figures and Tables

**Table 1:** The primer list.

Gene	Primer sequence (5′-3′)	Product length
GAPDH	5′ CTGGGCTACACTGAGCACC3′ 5′ AAGTGGTCGTTGAGGGCAATG3′	101
hsa_circRNA_104101 Divergent primer	F: 5′ GGAGCAACAGTAGAAAGTTCTCTA 3′ R: 5′ GGCACCTTCATCAGTAGTCATT 3′	56
hsa_circRNA_104101 Convergent primer	F: 5′ CATGGTAGCCACCCCAATGT 3′ R: 5′AATGGTGAGGAAACGCCGAT 3′	168

**Table 2:** The sequence information of siRNA.

Gene	siRNA sequence (5′-3′)
GAPDH	GCAACAGUAGAAAGUUCUCUATT
hsa_circRNA_104101 si-1 sense	UAGAGAACUUUCUACUGUUGCTT
hsa_circRNA_104101 si-1 antisense	GUAGAAAGUUCUCUACUUAAATT
hsa_circRNA_104101 si-2 sense	UUUAAGUAGAGAACUUUCUACTT
hsa_circRNA_104101 si-2 antisense	CAGUAGAAAGUUCUCUACUUATT
hsa_circRNA_104101 si-3 sense	UAAGUAGAGAACUUUCUACUGTT
hsa_circRNA_104101 si-3 antisense	GCAACAGUAGAAAGUUCUCUATT

**Table 3:** The primer list was used for real-time quantitative PCR.

Gene	Forward primer	Reverse primer
GAPDH	5′ CTGGGCTACACTGAGCACC3′	5′ AAGTGGTCGTTGAGGGCAATG3′
DSPP	5′TTTGGGCAGTAGCATGGGC3′	5′CCATCTTGGGTATTCTCTTGCCT3′
DMP1	5′CTCCGAGTTGGACGATGAGG3′	5′TCATGCCTGCACTGTTCATTC3′
OCN	5′ACCTCACCTTCCTCTACTTGG3′	5′TGGTGTCATTAGCCTTGCAG3′
hsa_circRNA_104101	5′GGAGCAACAGTAGAAAGTTCTCTA3′	5′GGCACCTTCATCAGTAGTCATT3′
hsa_circRNA_406763	5′ GTCTTTAGTCTTGGCAAAGGTGT3′	5′CAATGCATCAGAAAGGGAACA3′
hsa_circRNA_002161	5′TCCGGAGAACCAAACGGAAA3′	5′GAACTGTCCAAAGGCGGAAAA3′
hsa_circRNA_005044	5′CAAAGCAACGAGCTCTATGCC3′	5′CAAAGCAACGAGCTCTATGCC3′
hsa_circRNA_079813	5′TGGAACCTACAAGCAGCACC3′	5′TGCCATCAGGATTTCGATCTGT3′
hsa_circRNA_008336	5′TGGAACCTACAAGCAGGCAT3′	5′CTGGCAAAACCTGGGAAGAGA3′

## Data Availability

The datasets used and/or analyzed during the current study are available from the corresponding author on reasonable request.

## Supplementary Materials

Supplementary materials Additional file 1: Table S1: the expression profiles of upregulated circRNAs in induced and noninduced hDPSCs. Additional file 2: Table S2: the expression profiles of downregulated circRNAs in induced and noninduced hDPSCs. Additional file 3: certificate of English editing.
